# Effects of exoskeleton-assisted walking on bowel function in motor-complete spinal cord injury patients: involvement of the brain–gut axis, a pilot study

**DOI:** 10.3389/fnins.2024.1395671

**Published:** 2024-06-17

**Authors:** Xiaomin Hu, Jing Feng, Jiachun Lu, Rizhao Pang, Anren Zhang, Jiancheng Liu, Xiang Gou, Xingang Bai, Junyu Wang, Cong Chang, Jie Yin, Yunyun Wang, Hua Xiao, Qian Wang, Hong Cheng, Youjun Chang, Wenchun Wang

**Affiliations:** ^1^Department of Rehabilitation Medicine, The General Hospital of Western Theater Command, Chengdu, China; ^2^Department of Rehabilitation Medicine, Shanghai Fourth People’s Hospital, Tongji University School of Medicine, Shanghai, China; ^3^Chengdu Eighth People’s Hospital (Geriatric Hospital of Chengdu Medical College), Chengdu, China; ^4^College of Medicine, Southwest Jiaotong University, Chengdu, China; ^5^Care Alliance Jinchen Rehabilitation Hospital of Chengdu, Chengdu, China; ^6^School of Automation Engineering, University of Electronic Science and Technology of China, Chengdu, China; ^7^Sichuan Provincial Rehabilitation Hospital, Affiliated Rehabilitation Hospital of Chengdu University of T.C.M., Chengdu, China

**Keywords:** exoskeleton, spinal cord injury, constipation, bowel function, gut microbiota

## Abstract

Evidence has demonstrated that exoskeleton robots can improve intestinal function in patients with spinal cord injury (SCI). However, the underlying mechanisms remain unelucidated. This study investigated the effects of exoskeleton-assisted walking (EAW) on intestinal function and intestinal flora structure in T2-L1 motor complete paraplegia patients. The results showed that five participants in the EAW group and three in the conventional group reported improvements in at least one bowel management index, including an increased frequency of bowel evacuations, less time spent on bowel management per day, and less external assistance (manual digital stimulation, medication, and enema usage). After 8 weeks of training, the amount of glycerol used in the EAW group decreased significantly (*p <*0.05). The EAW group showed an increasing trend in the neurogenic bowel dysfunction (NBD) score after 8 weeks of training, while the conventional group showed a worsening trend. Patients who received the EAW intervention exhibited a decreased abundance of *Bacteroidetes* and *Verrucomicrobia*, while *Firmicutes*, *Proteobacteria*, and *Actinobacteria* were upregulated. In addition, there were decreases in the abundances of *Bacteroides*, *Prevotella*, *Parabacteroides*, *Akkermansia*, *Blautia*, *Ruminococcus 2*, and *Megamonas*. In contrast, *Ruminococcus 1*, *Ruminococcaceae UCG002*, *Faecalibacterium*, *Dialister*, *Ralstonia*, *Escherichia-Shigella*, and *Bifidobacterium* showed upregulation among the top 15 genera. The abundance of *Ralstonia* was significantly higher in the EAW group than in the conventional group, and *Dialister* increased significantly in EAW individuals at 8 weeks. This study suggests that EAW can improve intestinal function of SCI patients in a limited way, and may be associated with changes in the abundance of intestinal flora, especially an increase in beneficial bacteria. In the future, we need to further understand the changes in microbial groups caused by EAW training and all related impact mechanisms, especially intestinal flora metabolites.

**Clinical trial registration**: https://www.chictr.org.cn/.

## 1 Introduction

Spinal cord injury (SCI) is an increasingly important global health factor (GBD 2016 Traumatic Brain Injury and Spinal Cord Injury Collaborators, 2019). After SCI, neurogenic bowel dysfunction (NBD) is an almost inevitable condition; its main symptoms are faecal incontinence (FI) and constipation (Glickman and Kamm, 1996; Awad, 2011). According to reports, the prevalence of constipation in patients with SCI is between 56 and 80%, and FI occurs in 42–75% of patients (Tate et al., 2016; Qi et al., 2018). Approximately 78% of interviewees pointed out that bowel dysfunction was the main cause of quality of life impairment because bowel care (a large amount of time spent defaecating, compulsory drug use, digital stimulation for regular bowel movements, risk of faecal incontinence, persistent personal assistance needs, and other factors) interfered with their social life and personal relationships, preventing them from working away from home (Glickman and Kamm, 1996; Inskip et al., 2018). Improving intestinal function has been identified as one of the highest priorities for recovery in patients with SCI (including those with quadriplegia and paraplegia) (Anderson, 2004; Simpson et al., 2012; Lo et al., 2016). Generally, neurogenic bowel and subsequent symptoms in patients with motor-complete SCI were considered more severe than those in patients with motor-incomplete SCI (Vallès and Mearin, 2009). Research by Liu et al. showed that the risk of severe NBD in patients with American Spinal Cord Injury Association (ASIA) A was 12.8 times higher than that in patients with ASIA D (OR = 12.8, 95% CI 3.3–50.1) (Liu et al., 2010). These results are consistent with the finding that people with more severe injuries tend to have more serious bowel dysfunction (Vallès et al., 2006).

Individuals with SCI are susceptible to an imbalance in the intestinal flora. Using stool samples from mice with traumatic SCI, Kigerl et al. showed that traumatic SCI could cause intestinal diseases and that intestinal dysbiosis can disrupt functional recovery (Kigerl et al., 2016). According to reports, after SCI, the abundance of *Actinobacteria*, *Proteobacteria*, *Verrucomicrobia*, *Bacteroides*, *Blautia*, *Escherichia-Shigella*, *Akkermansia*, *Alistipes*, *Parabacteroides*, etc., increased significantly. In contrast, that of *Firmicutes*, *Faecalibacterium*, *Megamonas*, *Prevotella* 9, *Dialister*, *Roseburia*, etc., were significantly reduced (Gungor et al., 2016; Zhang et al., 2018; Bazzocchi et al., 2021; Du et al., 2021; Yu et al., 2021). Changes in the abundance of *Bifidobacterium*, *Lactobacillus*, *Bacteroides*, *Roseburia*, and other types of intestinal flora are related to constipation in patients with SCI (Zhu et al., 2014; Kim et al., 2015; Mancabelli et al., 2017; Zhang et al., 2021; Wang et al., 2023). Intestinal flora, such as *Bifidobacterium*, *Lactobacillus plantarum* P9, and *Latilactobacillus sakei*, have been shown to relieve constipation symptoms (Guo et al., 2022; Ma et al., 2023; Takeda et al., 2023). Studies have indicated an interactive relationship between constipation and intestinal flora. Constipation can lead to intestinal flora disorders, while intestinal flora dysfunction can also aggravate constipation symptoms, mainly characterised by increased pathogenic bacteria in the body and decreased dominant flora (Dimidi et al., 2017; Wortelboer et al., 2019; Fu et al., 2021). These results indicate that the microbiome may be a potential therapeutic target for constipation after SCI.

Physical activity, especially walking, as is universally acknowledged, can stimulate intestinal peristalsis in the able-bodied population (Peters et al., 2001; Bi and Triadafilopoulos, 2003; De Schryver et al., 2005; Hubscher et al., 2018). Exercise may modulate intestinal permeability, motility, stool transit time, and consistency (Sohail et al., 2019). Improvement in bowel function is more likely related to activity than an upright posture (Kwok et al., 2015). Studies have found that exercise interventions tend to increase the abundance of beneficial bacterial genera, such as *Blautia*, *Dialister*, and *Roseburia* (Quiroga et al., 2020). The exercise training of the exoskeleton system has been applied to rehabilitate paraplegic patients in recent years. The beneficial effects of exercise on improving intestinal function and intestinal flora have been widely confirmed in clinical medicine and exercise science (Allen et al., 2018; Gao et al., 2019; Resende et al., 2021).

Some studies have reported the effect of exoskeleton robots on patients with SCI, including ReWalk (ReWalk Robotics, Marlborough, MA, United States), Ekso (Ekso Bionics, Richmond, CA, United States), HAL (Cyberdyne, Tsukuba, Japan), and H-MEX (Hyundai Motor Company, Uiwang, Korea) Equipment (Gorgey et al., 2017; Baunsgaard et al., 2018; Chun et al., 2020; Gorman et al., 2021; Kim et al., 2021; Brinkemper et al., 2023). However, few studies have evaluated the relationship between microflora and the efficacy of EAW in treating constipation in individuals with SCI. In recent years, some scholars have suggested that the interactions between the microbiome, intestinal connectome, and brain connectome are established in the gut itself, meaning that if the input to the spinal cord or vagus nerve is cut off, the different components of ENS, mucosal immunity, and microbiome will still function (though not optimally; Macpherson et al., 2023). So is it possible that EAW training to treat constipation in SCI patients is related to intestinal flora? At the moment, there are no such reports. Therefore, in this study, we evaluated the role of EAW training in bowel function in patients with motor-complete SCI. The first to observe intestinal flora changes after EAW treatment of SCI, evaluate its correlation with intestinal function.

## 2 Materials and methods

### 2.1 Study design and ethics statement

A multicentre, randomised, single-blind, parallel-group clinical trial was conducted. The Institutional Review Boards of the General Hospital of Western Theater Command, Care Alliance Rehabilitation Hospital of Chengdu, and the Rehabilitation Hospital of Sichuan Province (2020ky011, 5, CKIL-2020028) approved the study protocol. This study was registered with the Chinese Clinical Trial Registry (ChiCTR2000035955). Informed consent was duly obtained from all participants. The clinician performed a physical examination for medical clearance at the start of the study to ensure safety.

### 2.2 Participants

Eligible individuals from three hospitals in Chengdu were recruited between October 2020 and July 2021. The inclusion criteria were as follows: (1) age: 20–60 years old; (2) classified American spinal injuries association impairment scale (ASIA) A or B, T2-L1 level of injury; (3) height between 1.55 m and 1.90 m, weight < 90 kg; (4) sitting balance scale ≥1, being able to stand with an assistive device but unable to walk independently; (5) no exposure to antibiotics, probiotics within the previous month; (6) able to sign for consent. Exclusion criteria were: (1) spasticity of any of the lower extremity muscles scored over 2 on the Modified Ashworth Scale, (2) uncontrolled hypertension (systolic blood pressure > 140 mmHg, diastolic blood pressure > 90 mmHg), and (3) complications (cardiopulmonary comorbidities, lower extremity decubitus, osteoporosis, past thrombosis/embolism, contractures or severe spasticity of the lower limb, and epilepsy).

Ten age-matched, healthy participants were recruited. The healthy participants were between 20 and 60 years old and had not taken antibiotics or probiotics during the month preceding enrolment. They also had no serious gastrointestinal disease or adverse hobbies such as long-term heavy drinking or smoking. Furthermore, they had no anxiety, depression, or other emotional disorders. All participants voluntarily participated in the experiment.

### 2.3 Randomisation and blinding

Patients were randomly categorised into EAW and conventional groups at a 1:1 ratio using computer-generated blocked random allocation schedules employing a simple randomisation method. The assignment sequences were placed in sealed, opaque, and sequentially numbered envelopes kept by individuals unrelated to the study. When the participants completed the preliminary assessment through the screening process, the envelope was opened to determine the group assignment. Only single-blind trials were conducted with assessors, data entry staff, and analysts because of the nature of the two interventions.

### 2.4 Interventions

**Figure 1:**
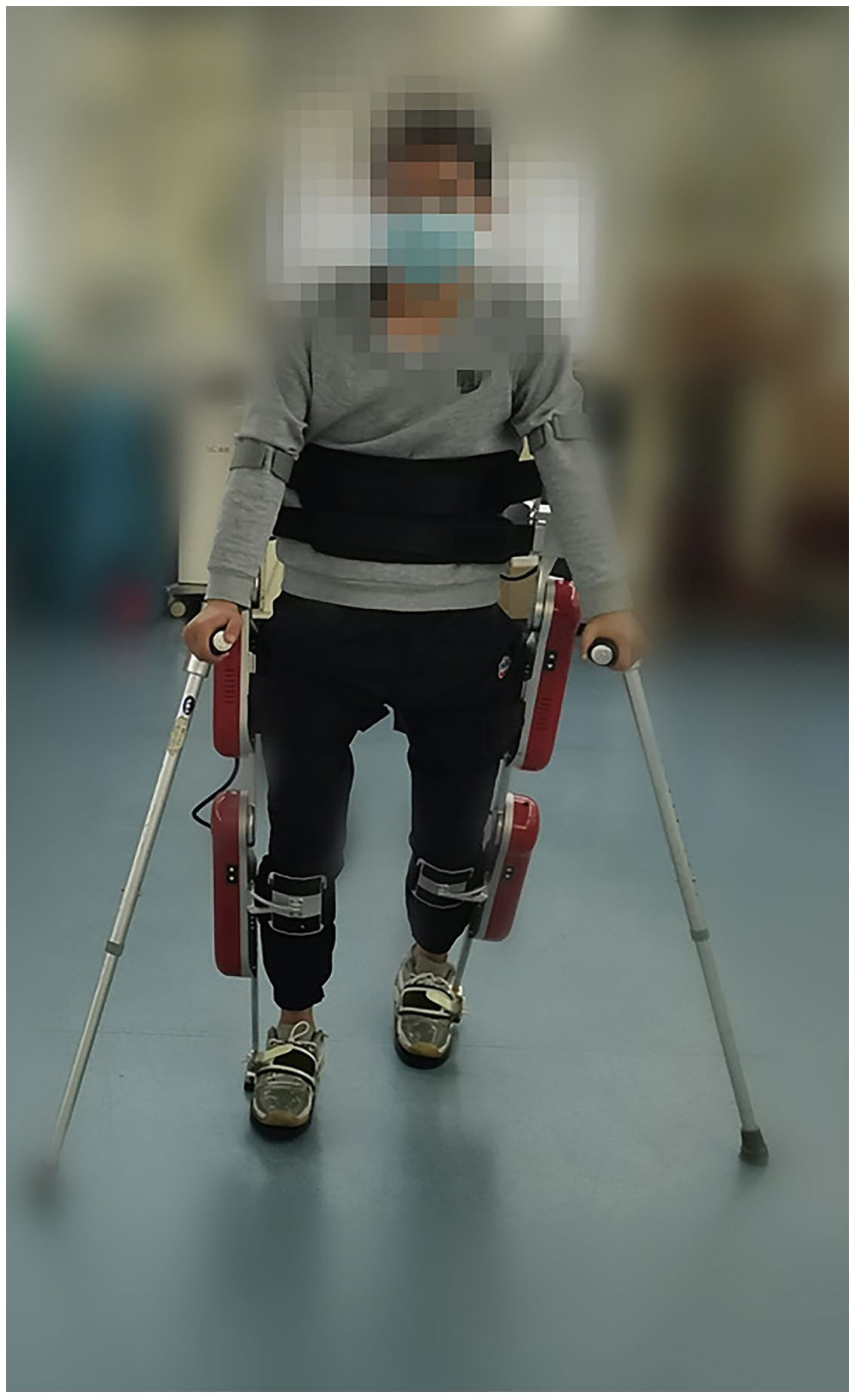
AIDER powered robotic exoskeleton (Buffalo Robot Technology Co. Ltd., Chengdu, China).

Master physicians and rehabilitation therapists assessed all participants with SCI according to their condition, and the corresponding routine rehabilitation training and medication regimens were drafted. The dietary habits and medications remained unchanged during the training period. All patients in the EAW group underwent AIDER powered robotic exoskeleton (Buffalo Robot Technology Co. Ltd., Chengdu, China) training 5 times a week for 8 weeks (Figure 1). Each session consisted of donning the device, checking vitals, performing a sit-to-stand, and walking for 40–50 min in the device, with occasional rest periods as needed. Details on the safety profile of EAW have been described in previous publications by Xiang et al. (2020). The conventional group only did regular rehabilitation training, including aerobic exercise and strength training. Qualified physiotherapists performed all the training sessions.

### 2.5 Bowel function assessments

Outcome indicators were collected and analysed at the baseline and end of the 8-week intervention. Two external individuals evaluated all outcome measurements.

Basic bowel management information was obtained from the International SCI dataset. The participants’ bowel movements in the past 4 weeks were analysed. Improvement was defined as a reduced bowel evacuation time, increased frequency of bowel movements, or reduced external assistance (including manual digital stimulation, medication use, and enema usage). Each of the five parameters is ranked from best to worst and assigned a value of 0, 1, 2, 3 in order to be converted into measurement data for statistical analysis.

1. Frequency of bowel evacuations: “every day (7 times a week)”, “2–6 times a week”, “once a week or less”.
2. Time spent on defecation: “0–30 min”, “31–60 min”, “more than 60 min”.
3. Manual digital stimulation needed: “none”, “less than once a week”, “once a week or more”, “once a day”.
4. Medication usage: “none”, “1–6 packs a week”, “One pack a day”.
5. Enema volume per defecation: the patients were inquired about the number of enemas applied each time.

The NBD score was based on a validated 10-item questionnaire on colorectal and anal dysfunction in individuals with SCI. The total score ranged from 0 (very mild NBD) to 47 (severe NBD).

### 2.6 16S rRNA analysis of faecal samples

Faecal samples were collected twice from patients with SCI: at baseline and after the eight-week intervention. However, samples from healthy participants were collected only once. In total, 42 faecal samples were collected. A disposable stool collection tube was used to collect the participants’ fresh faeces on the day of collection, and the samples were frozen at −80°C within 2 h to prevent repeated freezing and thawing.

The gDNA was purified using a Zymo Research BIOMICS DNA Microprep Kit (Cat # D4301). The integrity of all gDNA samples was verified by 0.8% agarose gel electrophoresis. The V4 region of the bacterial 16S rRNA gene was amplified with the common primer pair (515F,5´-GTGYCAGCMGCCGCGGTAA-3′; 806R, 5´-GGACTACH VGGGTWTCTAAT-3′). Each sample had three technical replicates. The PCR product was confirmed by gel electrophoresis (2% agarose gel) after loading the sample with a 6× loading buffer. Gel purification was performed using the Zymoclean Gel DNA Recovery Kit (D4008). After preparation, the libraries were quantified using a Qubit 2.0 Fluorometer (Thermo Scientific). Sequencing was performed using Illumina HiSeq (HiSeq Rapid SBS Kit v2, FC-402-4023, 500 cycles).

Subsequent bioinformatics operations were completed using QIIME2, while statistics and mapping were mainly completed using R6, Python, and Java. The following analyses were performed: OUT species annotation, species composition analysis, alpha/beta diversity, differential species analysis, and correlation analysis of the composition of the intestinal flora of the exoskeleton and conventional groups for different environmental factors.

### 2.7 Statistical analysis

SPSS 25.0 software was used for statistical analysis of the data, and *p* < 0.05 was considered to be statistically significant. The Shapiro–Wilk test was performed to determine the normality of the distribution. In the case of normal distribution, the data were expressed as mean ± SD. Independent samples t-test was used to compare the two groups of indicators, and paired samples t-test was used to compare the differences before and after the intervention. When the data did not conform to the normal distribution, the data were expressed as the median and interquartile distance (IQR). Wilcoxon Mann–Whitney U rank sum test was used to compare the two groups of indicators. Wilcoxon signed rank sum test was used to compare the differences before and after the intervention. Kruskal–Wallis H test was used to compare the alpha diversity of intestinal flora in multiple groups. ANOSIM analysis and STAMP analysis were used to compare the differences among intestinal flora groups. Spearman’s rank correlation analysis was used to evaluate the relationship between intestinal function severity and intestinal flora. All images were drawn using GraphPad Prism 8 and ChiPlot.

## 3 Results

### 3.1 Participants

**Figure 2:**
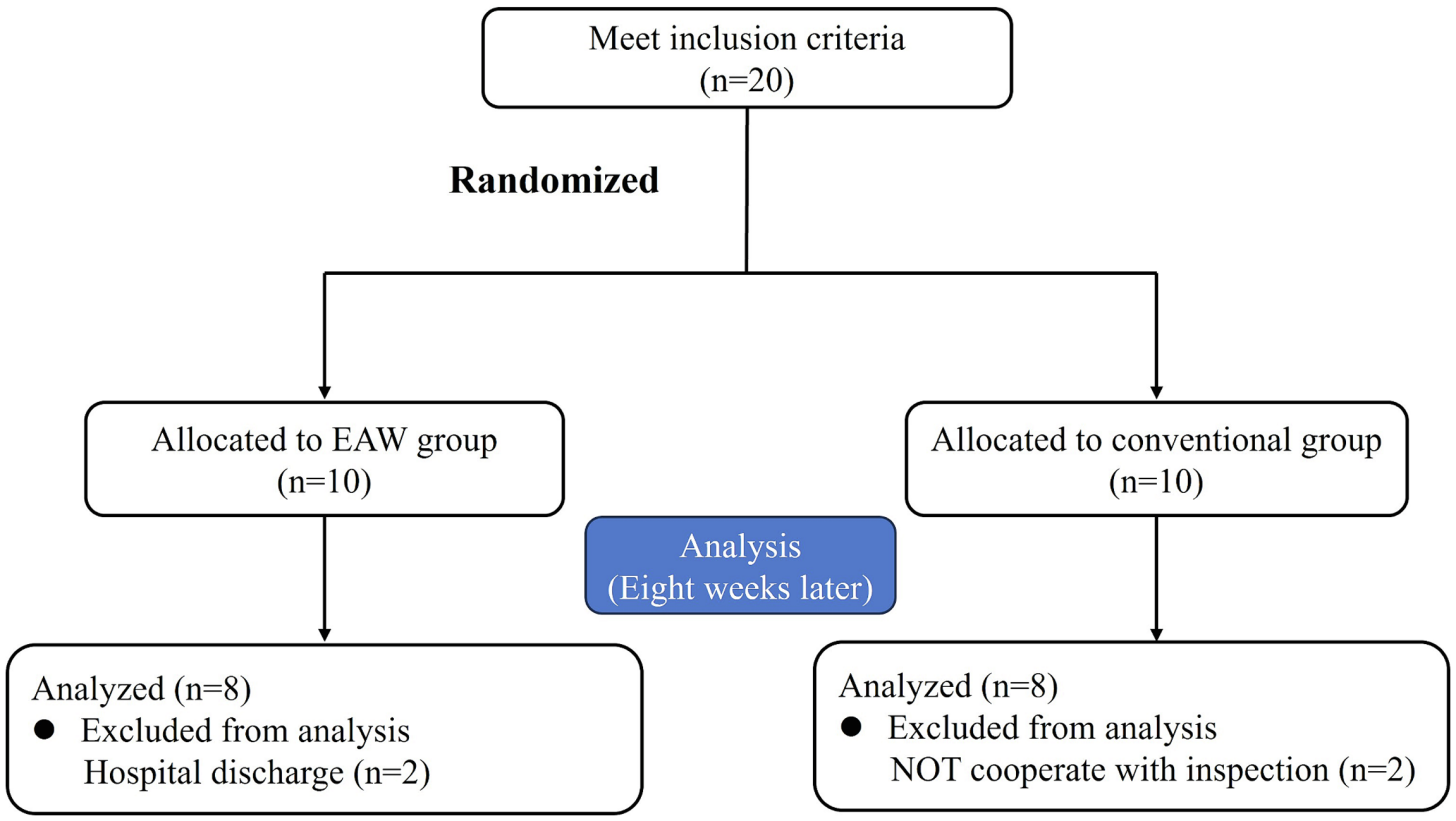
CONSORT diagram of enrolment of the study participants.

**Figure 3:**
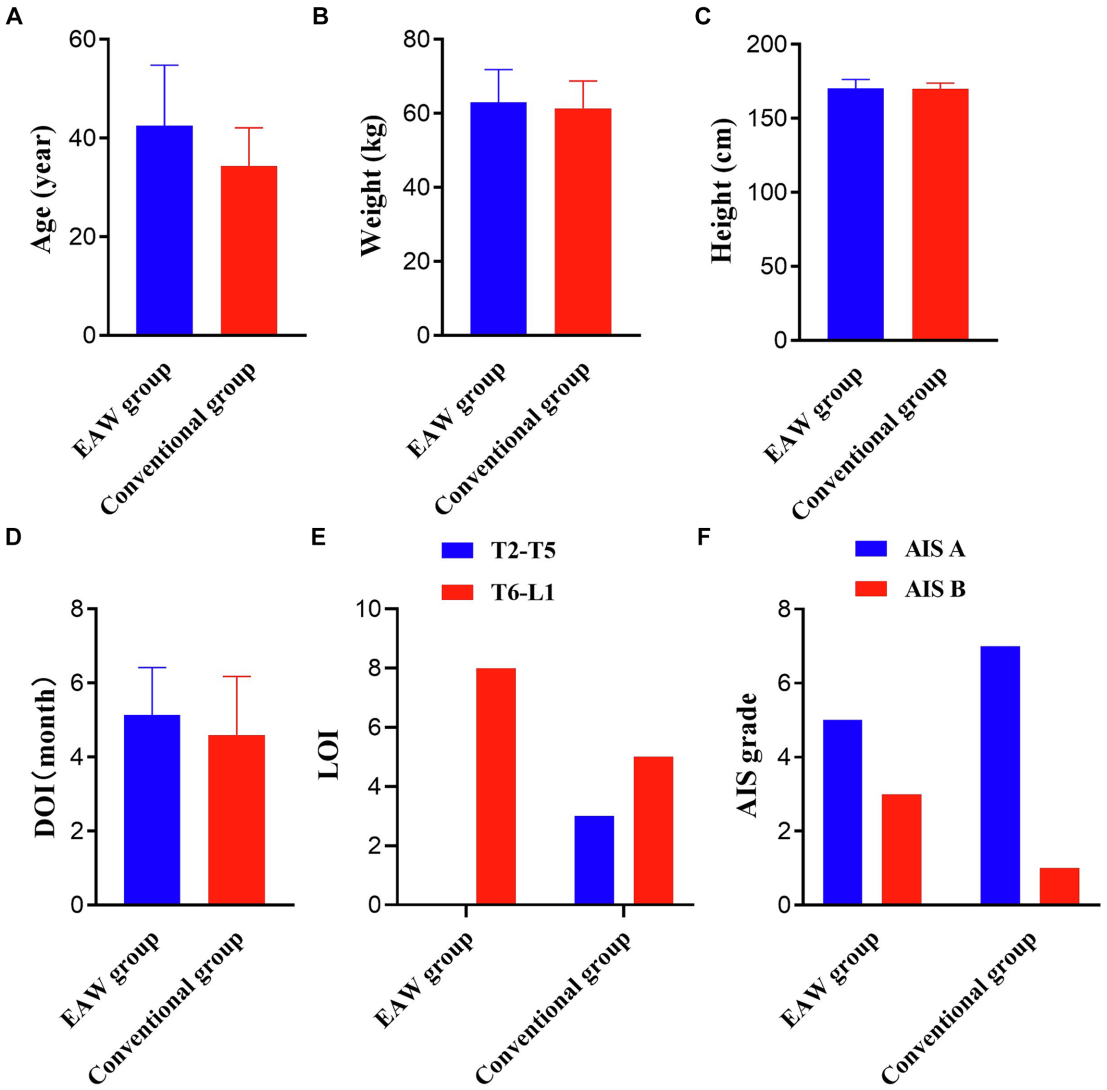
Results of the two groups at baseline. **(A)** Results pertaining age; **(B)** results pertaining weight; **(C)** results pertaining height; independent samples *t*-test; **(D)** results pertaining DOI; Wilcoxon Mann–Whitney U rank sum test; **(E)** results pertaining LOI; **(F)** results pertaining AIS grade; Fisher’s precision probability test; ^*^p < 0.05. DOI, duration of injury; LOI, level of injury; AIS, International Standards for Neurological Classification of SCI.

Twenty eligible patients were randomly categorised into the EAW (*n* = 10) and the conventional groups (*n* = 10). Two patients in the EAW group dropped out because of discharge, and two patients in the conventional group discontinued treatment for personal reasons. Consequently, these four participants were excluded from the data analysis. Figure 2 shows a CONSORT diagram. The demographic, clinical characteristics and baseline results are listed in Figure 3. There were no significant differences between the groups in age, weight, height, duration of injury, AIS grade, or injury level. These findings underscore the comparability of the EAW group and the conventional group.

### 3.2 General bowel management

**Figure 4:**
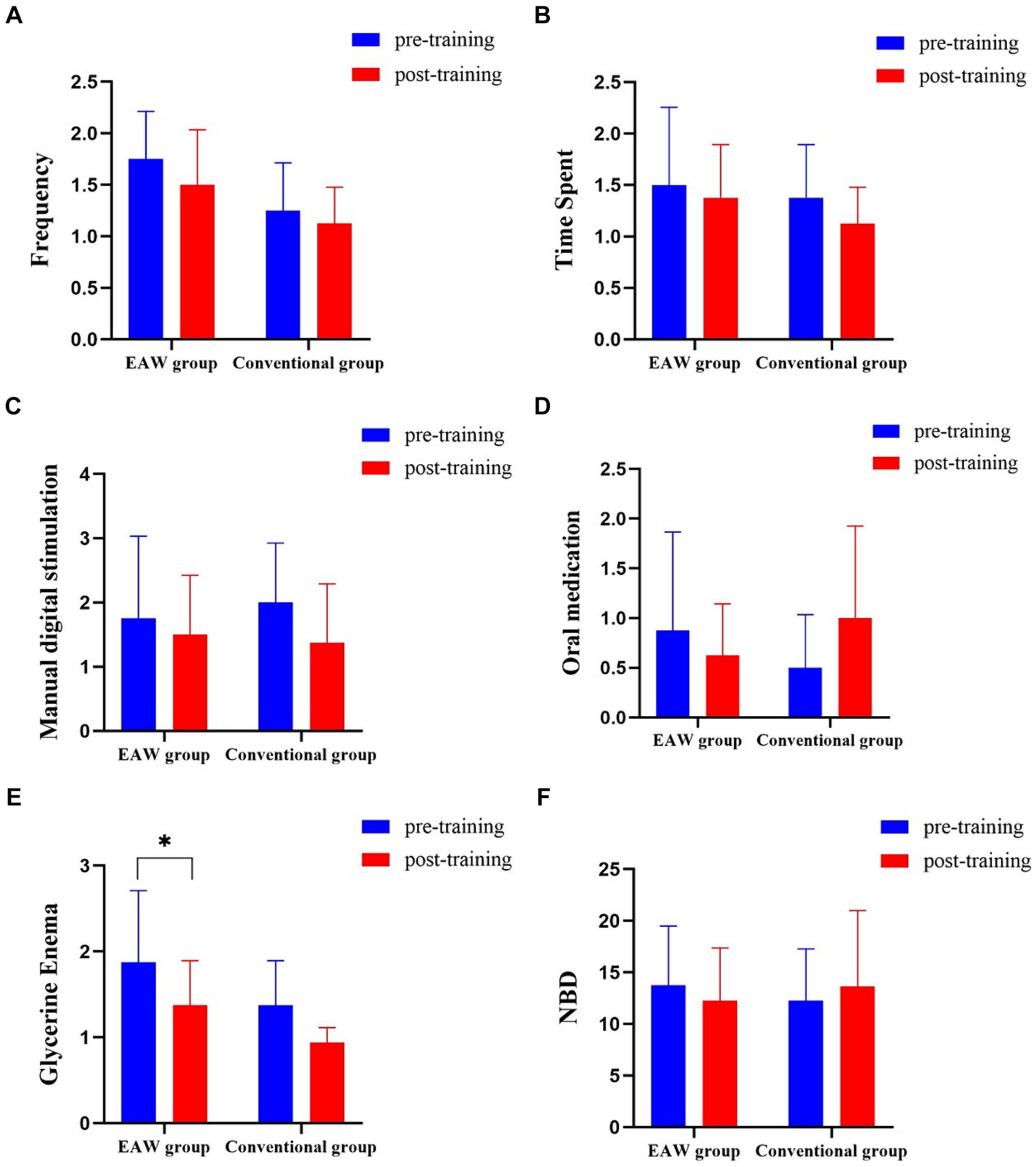
Results of the two groups at baseline and 8 weeks later. **(A)** Result of frequency; **(B)** result of time spent per bowel day; **(C)** result of manual digital stimulation for each bowel evacuation; **(D)** oral medication for each bowel evacuation; **(E)** Glycerine Enema each bowel evacuation; **(F)** result of NBD; the Wilcoxon Mann–Whitney U rank sum test was used for comparison between groups; The Wilcoxon signed rank sum test was used for intra-group comparison; ^*^p < 0.05. NBD, neurogenic bowel dysfunction.

Among the 16 participants in the study (EAW group = 8, conventional group = 8), the general bowel management information of nine participants was consistent with the NBD score results (Tables 1, 2). Among them, four participants (ID3, ID4, ID5, ID6) in the EAW group and two (ID12, ID13) in the conventional group showed improvement. However, two participants in the EAW group (ID2, ID8) and one in the conventional group (ID14) showed deterioration. In the remaining two participants (ID1, ID7) in the EAW group and five (ID9, ID10, ID11, ID15, ID16) conventional group patients, general bowel management information was inconsistent with the NBD score. In the five subproject analysis, no statistical difference was found between the two groups after 8 weeks of intervention. However, intra-group comparison showed that the number of glycerol enemas in the EAW group after intervention was lower than that before intervention, with statistical difference (z = −2.000, *p* = 0.046) (Figures 4A–E).

**Table 1:** Selected bowel items from the international SCI data set.

Group	ID	Frequency of bowel evacuations (# per week)		Time Spent per Bowel Day (# min)		Manual digital stimulation for each bowel evacuation		Oral medication for each bowel evacuation (# per week)		Glycerine Enema each bowel evacuation (# number/times)	
		Pre	Post	Pre	Post	Pre	Post	Pre	Post	Pre	Post
EAW group (n = 8)	1	2–6	2–6	0–30	0–30	Once or more times a week	Once or more times a week	1–6 Packs a week	1–6 Packs a week	2	1^*^
	2	2–6	2–6	0–30	31–60^†^	Once a week or less	Once a week or less	None	1–6 Packs a week^†^	2	2
	3	2–6	2–6	31–60	31–60	Once or more times a week	Once a week or less^*^	One pack a day	1–6 Packs a week^*^	3	2^*^
	4	2–6	7^*^	>60	31–60^*^	None	None	One pack a day	1–6 Packs a week^*^	3	2^*^
	5	2–6	7^*^	31–60	0–30^*^	Once a day	Once or more times a week	One pack a day	1–6 Packs a week^*^	2	1^*^
	6	2–6	7^*^	0–30	0–30	None	Once a week or less	None	None	1	1
	7	7	7	0–30	0–30	Once a day	Once a day	None	None	1	1
	8	7	2–6^†^	0–30	0–30	Once a day	Once or more times a week	None	None	1	1
Conventional group (*n* = 8)	9	7	7	0–30	0–30	Once or more times a week	Once or more times a week	1–6 Packs a week	1–6 Packs a week	1	1
	10	7	7	0–30	0–30	Once a day	Once or more times a week	None	1–6 Packs a week^†^	2	1^*^
	11	7	7	31–60	31–60	Once or more times a week	Once or more times a week	None	None	2	0.5^*^
	12	7	7	0–30	0–30	Once a day	Once or more times a week	1–6 Packs a week	None^*^	1	1
	13	2–6	7^*^	31–60	0–30^*^	None	None	None	None	1	1
	14	7	2–6^†^	0–30	0–30	Once or more times a week	Once or more times a week	1–6 Packs a week	One pack a day^†^	2	1^*^
	15	7	7	0–30	0–30	Once or more times a week	Once a week or less^*^	None	One pack a day^†^	1	1
	16	2–6	7^*^	31–60	0–30^*^	Once or more times a week	None^*^	1–6 Packs a week	One pack a day^†^	1	1

^*^Improved compared with before treatment; ^†^Worsened compared with before treatment.

**Table 2:** NBD score of all involved patients.

Groups	ID	Neurogenic Bowel Dysfunction Score (points/47)	
		Pre	Post
EAW group (*n* = 8)	1	17	17
	2	6	10
	3	22	14
	4	12	7
	5	14	10
	6	6	5
	7	19	15
	8	14	20
Conventional group (*n* = 8)	9	10	18
	10	11	19
	11	20	20
	12	19	11
	13	6	2
	14	10	21
	15	8	4
	16	14	14

The number of enemas used by ID1 was changed from 2 to 1 each time, and the NBD score remained unchanged. ID7’s NBD score was changed from 14 to 20. Among the five participants in the conventional group, the five bowel management parameters of ID9 remained unchanged, and the NBD score increased by 8 points (10–18). The number of enemas for ID11 was reduced from two to 0.5, but the NBD score remained at 20 points. ID15 and ID16 changed with the help of external functions; the NBD score of the former dropped by 4 points (8–4), and that of the latter remained unchanged.

Figure 4F presents the NBD scores of the participants who were treated with EAW training, showing a downward trend (13.75 ± 5.73, 12.25 ± 5.12) after 8 weeks of treatment. However, the conventional group had an upward trend (12.25 ± 5.04, 13.63 ± 7.35), but neither trend showed significant differences.

### 3.3 Gut microbiota composition

**Figure 5:**
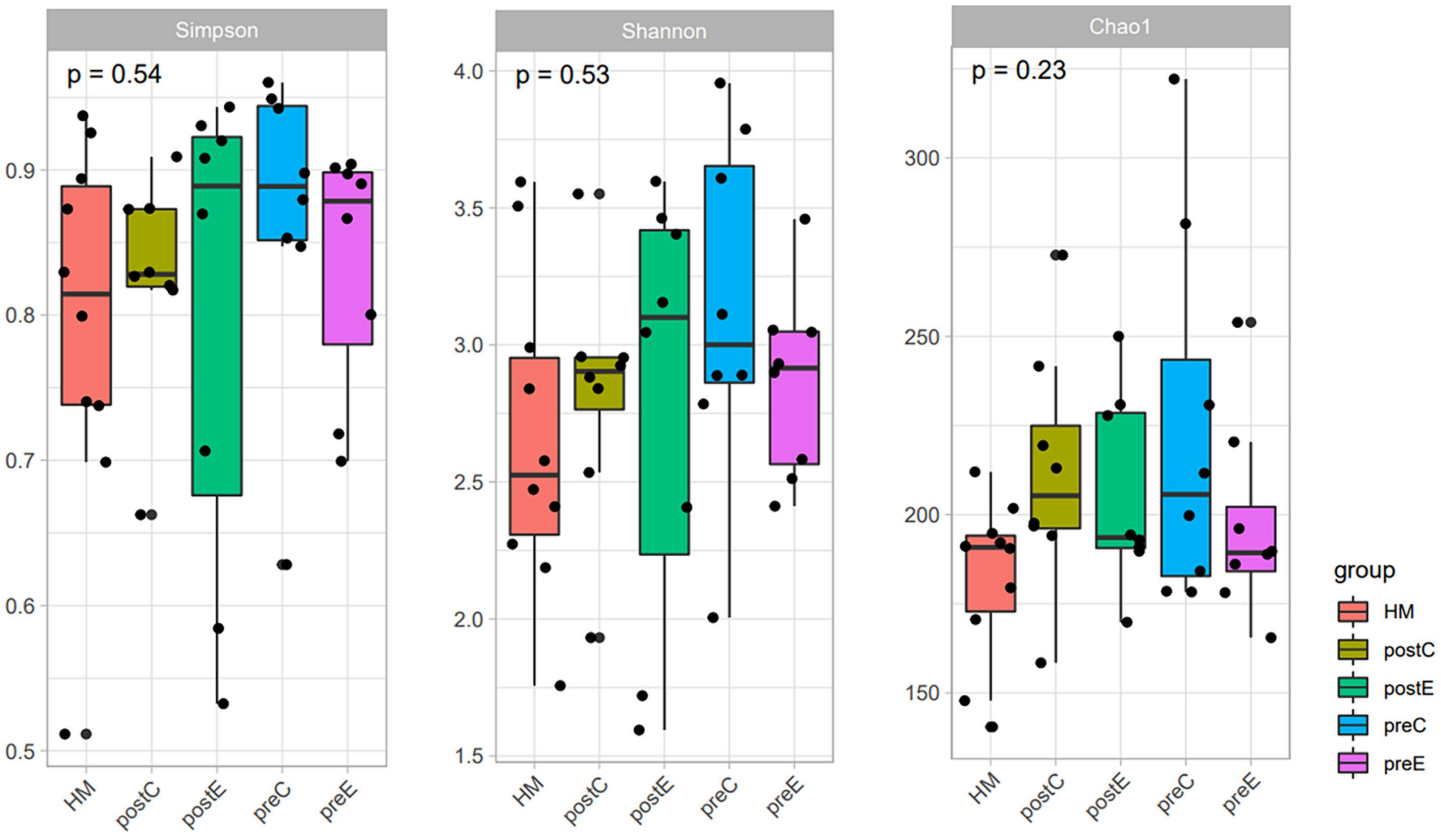
Results of Chao1, Shannon, and Simpson index; The abundance (assessed by Chao Index) and diversity (assessed by Shannon and Simpson index) of intestinal flora in the two groups were compared based on ASV levels. Kruskal–Wallis H test; ^*^p < 0.05.

**Figure 6:**
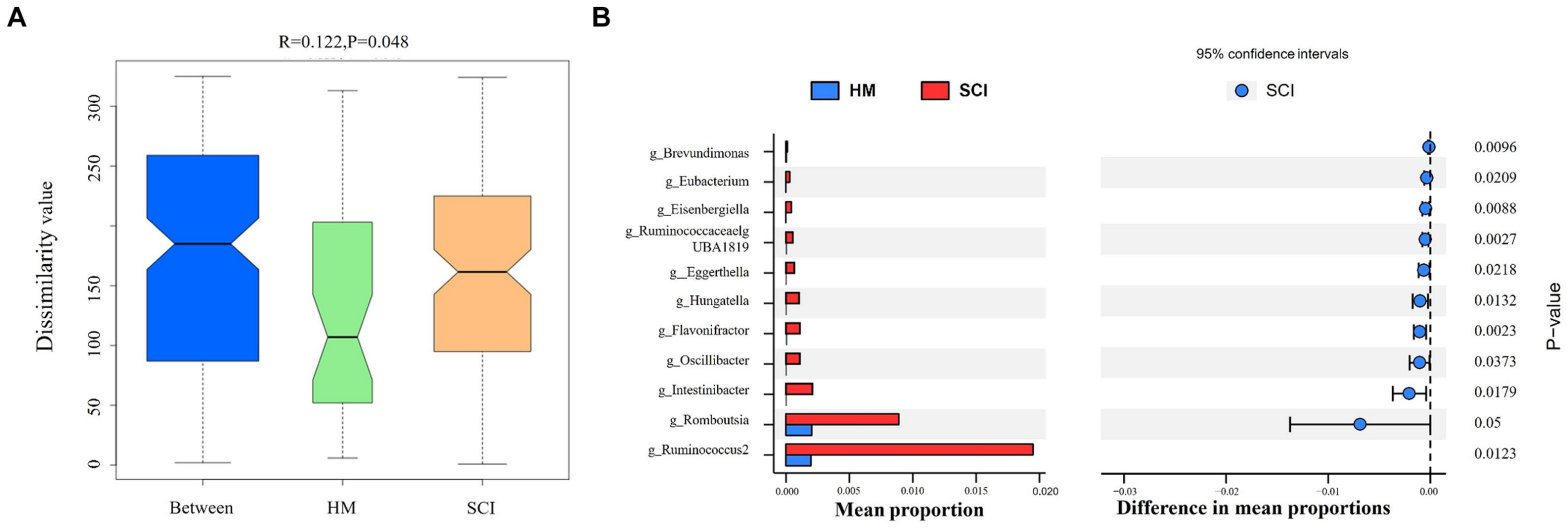
**(A)** ANOSIM analysis, reflecting beta diversity, was used to compare differences in intestinal flora community structure between SCI patients and healthy men; **(B)** STAMP analysis was used to compare the difference in intestinal flora species abundance between SCI patients and healthy people.

The top five bacterial phyla were Bacteroidetes, Firmicutes, Proteobacteria, Actinobacteria, and Verrucomicrobia. The top five genera were *Bacteroides*, *Ruminococcaceae* 1, *Prevotella* 9, UCG-002, and *Parabacteroides*. Rarefaction and rank-abundance curves showed that the amount of data sequenced in the experiment was reasonable. No significant difference in alpha diversity (Chao1 or Shannon or Simpson index, etc.) was observed between healthy men and SCI patients, and 8 weeks of intervention (EAW/conventional rehabilitation training) did not have a significant effect on the abundance of microbiota in patients with SCI and constipation (Figure 5). ANOSIM analysis showed a significant difference in beta diversity between SCI patients and healthy men (ANOSIM, *p* < 0.05; Figure 6A).

STAMP analysis showed that SCI leads to an imbalance in the intestinal flora. No significant variation was detected at the phylum level; however, the abundances of *Brevundimonas, Eubacterium, Eisenbergiella, Ruminococcaceae-UBA1819, Eggerthella, Hungatella, Flavonifractor, Oscillibacter, Intestinibacter, Romboutsia, Ruminococcus 2* were significantly higher than those in the healthy male group (*p* < 0.05) at the genus level (Figure 6B). EAW intervention partially improved the intestinal flora imbalance in patients with SCI.

**Figure 7:**
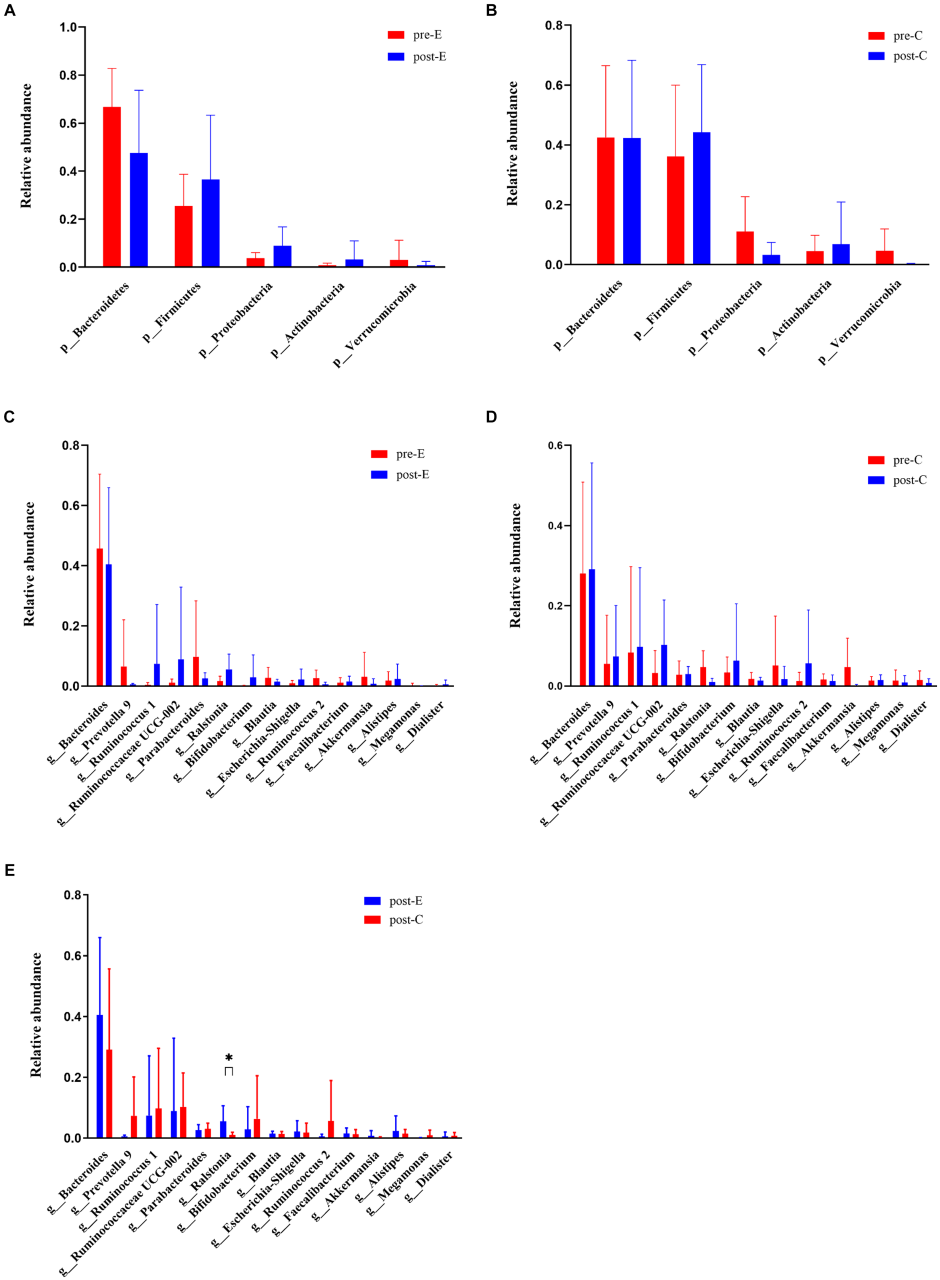
Changes in phylum levels and top 15 genus levels of intestinal flora in both groups at baseline and 8 weeks later. **(A)** Changes in phylum levels in the EAW group at baseline and after 8 weeks of intervention; **(B)** changes in phylum levels in the Conventional group at baseline and after 8 weeks of intervention; **(C)** changes in top 15 genus levels in the EAW group at baseline and after 8 weeks of intervention; **(D)** changes in top 15 genus levels in the conventional group at baseline and after 8 weeks of intervention; **(E)** the difference of top 15 genus levels between the two groups after 8-week intervention was compared. ^*^p < 0.05. HM, healthy men; Pre- or Post-C, pre- or post- conventional intervention; Pre- or Post-E, pre- or post-EAW intervention.

After EAW treatment, the abundance of phylum Bacteroidetes (genus *Bacteroides*, genus *Prevotella 9*, genus *Parabacteroides*) and phylum Verrucomicrobia (genus *Akkermansia*) decreased at the phylum level and the top 15 genera levels. However, the phylum Firmicutes (genus *Ruminococcus 1*, genus *Ruminococcaceae UCG002*, genus *Faecalibacterium*, genus *Dialister*), the phylum Proteobacteria (genus *Ralstonia*, genus *Escherichia-Shigella*), and the phylum Actinobacteria (genus *Bifidobacterium*) upregulated. Additionally, the abundance of the genus *Alistipes* upregulated, and the genus *Blautia*, genus *Ruminococcus 2*, and genus *Megamonas* dropped. The improvement in EAW on *Ralstonia* abundance was significantly greater than that in the conventional group, and *Dialister* abundance increased significantly in individuals treated with EAW at 8 weeks. In addition, *Ruminococcus 1*, *Ruminococcaceae UCG-002,* and *Bifidobacterium* content showed an upward trend, whereas the abundances of *Blautia*, *Akkermansia,* and *Megamonas* showed a downward trend (Figure 7).

**Figure 8:**
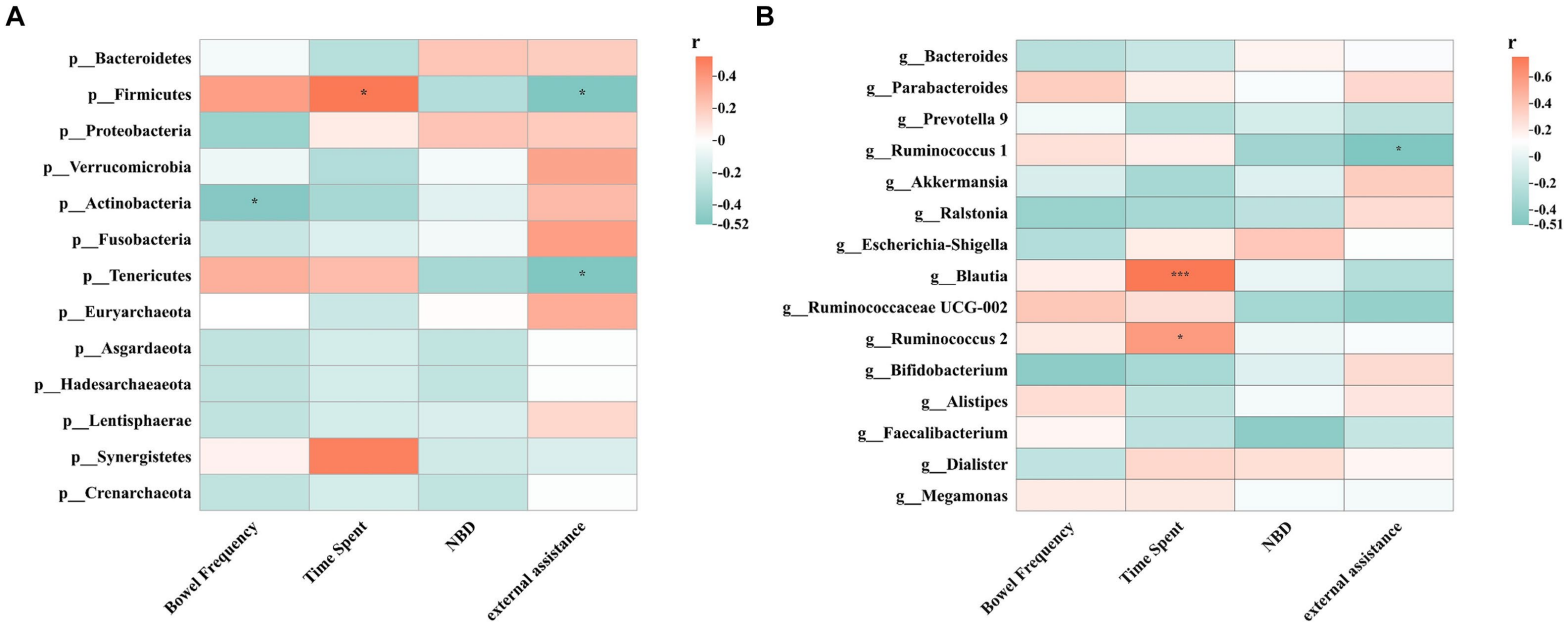
Spearman correlations between intestinal flora and clinical indicators including NBD, external assistance, time spent and bowel frequency. **(A)** Spearman correlation between phylum levels and clinical indicators; **(B)** Spearman correlation between top 15 genus levels and clinical indicators; ^*^p < 0.05, ^**^p < 0.01, ^***^p < 0.001.

Spearman’s correlation test evaluated the relationship between gut microbes and various indicators (Figure 8). The results showed that Firmicutes abundance was positively correlated with defaecation time (Spearman *r* = 0.523, *p* = 0.038), while negatively correlated with external assistance (Spearman *r* = −0.521, *p* = 0.038). Actinobacteria abundance negatively correlated with defaecation frequency (Spearman *r* = −0.500, *p* = 0.049). Tenericutes abundance was negatively correlated with external assistance (Spearman *r* = −0.500, *p* = 0.049). Among the top 15 genera, *Ruminococcus 1* abundance was negatively correlated with external assistance (Spearman *r* = −0.513, *p* = 0.042). *Blautia* abundance was positively correlated with defaecation time (Spearman’s *r* = 0.752, *p* = 0.000), and *Ruminococcus 2* abundance positively correlated with defaecation time (Spearman’s *r* = 0.591, *p* = 0.016).

## 4 Discussion

SCI is a sudden and catastrophic event. The severity of the injury and functional recovery vary among individuals. The most important determinant of prognosis is whether the injury is complete or incomplete. Up to 25% of complete injuries are incomplete within the first year after injury. However, functional motor recovery (e.g., weight-bearing and walking) at the distal end of the injury area after complete motor SCI is rare (Angeli et al., 2018; Fouad et al., 2021). This is because the motor center is difficult to activate in patients with complete motor spinal cord injury and may exhibit a more pronounced activation effect when using epidural stimulation, but this technology is still in the promotion stage, and the combined effect of physical rehabilitation needs to be further improved (Côté et al., 2017; Gorgey et al., 2021). AIS A and B are classified as motor-complete injuries with more serious dysfunction and life restrictions. SCI changes intestinal peristalsis and sphincter control, and the loss of mobility and movement flexibility makes intestinal dysfunction a major life-limiting factor (Lynch and Frizelle, 2006). Powered robot exoskeletons have low energy consumption, intelligence, efficiency, and power, enabling patients with complete motor SCI to achieve therapeutic walking and increase the possibility of functional recovery.

After the 8-week intervention, five participants (62.5%) in the EAW group described improvement in at least one or more items of bowel function management, compared to only three participants (37.5%) in the conventional group. At the same time, the number of glycerol enemas in EAW group decreased significantly after the intervention (*p <* 0.05). In addition, as our results from NBD scores indicate, the EAW group exhibited a downward trend in the NBD score, while the conventional group showed an overall upward trend. A lower NBD score indicated better bowel function. Although there was no statistically significant improvement in intestinal function before and after the intervention in either group, patients in the EAW group were more likely to benefit from the training. Our results suggest EAW improved bowel function in patients with SCI to a certain extent, but it did not have the desired effect. Consistent with previous studies, among spinal cord injury patients who underwent 25–63 ReWalk sessions, more than 50% of participants experienced a shortened time to defaecation, a decrease in bowel accidents, and a reduction in the frequency of laxative, stool softening use, or both, and overall bowel satisfaction improved (Chun et al., 2020; Brinkemper et al., 2023). Gorman et al. reported in a later large-scale randomised controlled crossover study that EAW training had a positive impact on approximately one-quarter of the participants in terms of bowel function and management, but there was no statistical difference in the frequency and duration of bowel movements, and the magnitude of the impact was not as significant as initially assumed (Gorman et al., 2021). However, contrary results were obtained by Baunsgaard et al., who found that intestinal function did not improve in patients with chronic spinal cord injury, but improved in patients with recent injuries after 24 sessions of robotic exoskeleton gait training, which may be related to different study design, geography, etc. (Baunsgaard et al., 2018). Notably, the patients involved in this study were injured for a short period. The reported intestinal function improvement effect of the EAW in this study may be partly attributed to the early self-recovery of patients with newer SCI. The SCI subjects included in this study were all motor-complete injury, with more serious intestinal function involvement and slower functional recovery, which may be related to the inobvious improvement. In addition, due to the small sample size, most of the functional changes were not statistically significant, and the sample size will be increased in the future and more rigorous protocols will be adopted to enhance the reliability of the study.

After SCI, the influence of the spinal cord on autonomic nervous system (ANS) is destroyed, which leads to sympathetic passivation and parasympathetic innervation, resulting in multiple organ disorders (Henke et al., 2022). The sympathetic nervous system (SNS) originates from the T1-L2 spinal segment, sympathetic preganglionic neurons (SPN) from T9-T12 innervate the ascending colon, large intestine, and small intestine, and SPN from T12-L2 innervate the descending colon and rectum (Browning and Travagli, 2014; Wulf and Tom, 2023). Thus, both lower thoracic and lumbar spinal cord injuries involve the sympathetic nervous system, leading to neurogenic intestinal dysfunction characterized by constipation, abnormal excretory reflexes, and decreased colonic contractions. Only one subject with L1 injury was included in this trial, and his bowel function was not better than that of all other patients with thoracic injury. In the future, patients with spinal cord injury at different segments will be included for stratified analysis of intestinal function.

The tens of trillion microbes in the intestine regulate the metabolism of the host, and physical exercise seems to increase the abundance of some healthy microbes (Barton et al., 2018; Lensu and Pekkala, 2021; Moitinho-Silva et al., 2021). In our study, EAW altered the gut microbiota of SCI patients, with increased abundance of *Dialister* (*p* < 0.05), *Faecalibacterium*, *Alistipes* and *Bifidobacterium*. Previous studies have reported that *Faecalibacterium* are correlated with faster colonic transit (Parthasarathy et al., 2016), and *Dialister* is reduced in SCI patients, which may aggravate the symptoms of NBD (Zhang et al., 2018). *Dialister* and *Faecalibacterium* belong to Firmicutes. In the correlation analysis, we found that the abundance of *Firmicutes* negatively correlated with external assistance. *Faecalibacterium*, *Dialister* and *Bifidobacterium*, is a producer of butyrate, a short-chain fatty acid. Previous studies have confirmed that butyrate induces the release of serotonin, thereby activating the maturation of the enteric nervous system, restoring colon movement, and relieving constipation (Reigstad et al., 2015; Yano et al., 2015; Ge et al., 2017). Although the specific mechanisms by which the gut microbiota regulates 5-HT synthesis have not been explored, gut microbiota-derived metabolites, such as tryptophan, secondary bile acids, and short-chain fatty acids (SCFAs), are all involved in triggering 5-HT biosynthesis in EC cells (Sun et al., 2018; Rosario et al., 2021; Bai et al., 2022). In addition, it has been found that exercise can increase the concentration of butyricogenes in mice and humans to enrich the concentration of SCFAs. SCFAs can enhance fecal water content and colonic contractility, and reduced colon transit time (Wang et al., 2017; Zhao et al., 2018). It has been reported that *Alistipes* is negatively correlated with defecation time (Zhang et al., 2018). No such relationship was found in this study, but *Alistipes* increased slightly and defecation time decreased slightly after EAW training. In addition, *Alistipes* is a producer of propionate and acetate (El-Salhy, 2023), Butyrate has been proved to induce the expression of AHR and CYP1A1, while propionate can only induce the expression of AHR (Korecka et al., 2016). Some studies have shown that AHR expressed by intestinal cells can enhance intestinal motility function and form an automatic regulatory feedback loop with CYP1A1, which can metabolize AHR ligands and terminate AHR signal transduction, while the expression of CYP1A1 is dependent on AHR (Korecka et al., 2016; Schiering et al., 2017; Obata et al., 2020). Increasing the concentration of butyrate and propionate can activate the expression of AHR and CYP1A1, which can increase intestinal peristalsis and improve constipation. In short, the increase of beneficial bacteria such as *Faecalibacterium*, *Dialiste*r, *Bifidobacterium* and Alistipes may be one of the potential reasons for EAW to alleviate intestinal dysfunction. We will also confirm the role of intestinal flora and its metabolites SCFAs in future studies.

It is worth noting that we found that the level of *Blautia* in the two groups has declined, and it is positively related to the defecation time. This is inconsistent with the increase in 12-week exercise training reported by Quiroga et al. (2020). In addition, *Megamonas* has been reported to have beneficial properties as its reduction exacerbates NBD symptoms (Zhang et al., 2018), whereas our study found that *Megamonas* showed a downward trend after EAW intervention. One reason for these inconsistencies may be that *Blautia* and *Megamonas* are not particularly sensitive to athletic training. It seems to take a specific intensity or amount of exercise to improve.

The abundance of *Proteobacteria* is significantly decreased in patients with constipation (Guo et al., 2020). We found that the relative abundance of *Proteobacteria* increased in the EAW group but decreased in the conventional group. However, at baseline, *Proteobacteria* abundance in the EAW group was lower than that in healthy men, while that in the conventional group was higher. Munukka et al. reported that 6 weeks of endurance training attenuated *Proteobacteria* in overweight women (Munukka et al., 2018). So the hypothesis is that exercise might adjust *Proteobacteria* to a healthy level rather than cause an absolute increase or decrease. These results indicated that *Proteobacteria* may be particularly sensitive to intervention. *Ralstonia* belongs to the phylum *Proteobacteria* and is inversely associated with the severity of antipsychotic-related constipation in patients aged 18–23 (Zheng et al., 2021). In addition, the abundance of *Ralstonia* was reported to be severely reduced in the rectal samples of patients with irritable bowel syndrome compared to healthy controls, which seems to indicate that the reduction of *Ralstonia* has a negative effect on bowel function (Loranskaia et al., 2013). In this study, the abundance of *Ralstonia* increased significantly in the EAW group compared with that of the control group. The beneficial effects of the EAW training may be mediated in part by *Ralstonia*, but there are few reports about *Ralstonia* and more studies are needed to confirm it.

Remarkably, the abundance of *Verrucomicrobia* decreased in both groups, with *Akkermansia* being one of the main representatives of *Verrucomicrobia*. This study found that participants with a high abundance of *Akkermansia* at baseline showed a more pronounced reduction in the conventional group, although *Akkermansia* also decreased in the EAW group. According to previous reports, the aggravation of constipation is related to the increase in *Akkermansia* (Cao et al., 2017; Fu et al., 2021; Ma et al., 2023). Surprisingly, an increasing number of studies have shown that *Akkermansia* plays a key role in keeping the gastrointestinal tract intact and is considered the next generation of probiotics (Everard et al., 2013; Li et al., 2016; Munukka et al., 2018; Macchione et al., 2019; Wegierska et al., 2022). Exercise has opposite effects on *Akkermansia* in different populations (Munukka et al., 2018; Mokhtarzade et al., 2021). As microbes can have multiple effects on humans, it was difficult to determine whether the increase in the abundance of *Akkermansia* after SCI was negative or positive. It’s not clear how exercise affects *Akkermansia*, but at least it can be regulated.

## 5 Study limitations

First of all, the sample quantity of this experiment is very small, so it is necessary to increase the amount of samples to improve the statistical power, and to determine whether there is any difference in intestinal function recovery of patients with different injury segments by EAW. In addition, Gorman’s experiments showed that men’s intestinal function improved more than women’s after using EAW. The SCI patients in this study were all men, and whether gender was a factor remains to be investigated. Finally, the link between changes in gut microbiota composition and exercise regulation of intestinal function has not been fully demonstrated, and whether SCFAs play a role in it, more experiments are needed to support this relationship. The results of our pilot study must be confirmed in a larger scale in the future.

## 6 Conclusion

The implementation of 40 exoskeleton-assisted walking programs in patients with spinal cord injury who were unable to walk provided a limited degree of improvement in intestinal function. The effect of EAW training on intestinal function may be related to changes in the abundance of intestinal flora, especially the increase of beneficial bacteria. However, further research is needed to fully understand the changes in microbial groups caused by EAW training, and all their associated effects, especially gut microbiota metabolites. At the same time, AIDER robots with affordable exoskeletons need to be applied in the home to observe the impact of long-term maintenance training on SCI patients.

## Data availability statement

The datasets used and analysed in this study are available from the corresponding author upon reasonable request.

## Ethics statement

The studies involving humans were approved by the Ethics Committee of the General Hospital of Western Theater Command, Care Alliance Jinchen Rehabilitation Hospital of Chengdu, and the Rehabilitation Hospital of Sichuan Province. The studies were conducted in accordance with the local legislation and institutional requirements. The participants provided their written informed consent to participate in this study.

## Author contributions

XH: Writing – original draft, Investigation, Methodology. JF: Writing – original draft. JLu: Data curation, Writing – review & editing. RP: Supervision, Writing – review & editing. AZ: Methodology, Supervision, Writing – review & editing. JLi: Data curation, Writing – review & editing. XG: Investigation, Writing – review & editing. XB: Investigation, Writing – review & editing. JW: Supervision, Writing – review & editing. CC: Investigation, Writing – review & editing. JY: Investigation, Writing – review & editing. YW: Investigation, Writing – review & editing. HX: Methodology, Writing – review & editing. QW: Supervision, Writing – review & editing. HC: Supervision, Writing – review & editing. YC: Supervision, Writing – review & editing. WW: Methodology, Project administration, Supervision, Writing – review & editing.

## References

[ref1] Allen J. M. Mailing L. J. Niemiro G. M. Moore R. Cook M. D. White B. A.. (2018). Exercise alters gut microbiota composition and function in lean and obese humans. Med. Sci. Sports Exerc. 50, 747–757. doi: 10.1249/MSS.0000000000001495, PMID: 29166320

[ref2] Anderson K. D. (2004). Targeting recovery: priorities of the spinal cord-injured population. J. Neurotrauma 21, 1371–1383. doi: 10.1089/neu.2004.21.1371, PMID: 15672628

[ref3] Angeli C. A. Boakye M. Morton R. A. Vogt J. Benton K. Chen Y.. (2018). Recovery of over-ground walking after chronic motor complete spinal cord injury. N. Engl. J. Med. 379, 1244–1250. doi: 10.1056/NEJMoa1803588, PMID: 30247091

[ref4] Awad R. A. (2011). Neurogenic bowel dysfunction in patients with spinal cord injury, myelomeningocele, multiple sclerosis and Parkinson’s disease. World J. Gastroenterol. 17, 5035–5048. doi: 10.3748/wjg.v17.i46.5035, PMID: 22171138 PMC3235587

[ref5] Bai J. Cai Y. Huang Z. Gu Y. Huang N. Sun R.. (2022). Shouhui Tongbian capsule ameliorates constipation via gut microbiota-5-HT-intestinal motility axis. Biomed. Pharmacother. 154:113627. doi: 10.1016/j.biopha.2022.113627, PMID: 36058152

[ref6] Barton W. Penney N. C. Cronin O. Garcia-Perez I. Molloy M. G. Holmes E.. (2018). The microbiome of professional athletes differs from that of more sedentary subjects in composition and particularly at the functional metabolic level. Gut 67, gutjnl-2016-313627–gutjnl-2016-313633. doi: 10.1136/gutjnl-2016-313627, PMID: 28360096

[ref7] Baunsgaard C. B. Nissen U. V. Brust A. K. Frotzler A. Ribeill C. Kalke Y. B.. (2018). Exoskeleton gait training after spinal cord injury: an exploratory study on secondary health conditions. J. Rehabil. Med. 50, 806–813. doi: 10.2340/16501977-2372, PMID: 30183055

[ref8] Bazzocchi G. Turroni S. Bulzamini M. C. D’Amico F. Bava A. Castiglioni M.. (2021). Changes in gut microbiota in the acute phase after spinal cord injury correlate with severity of the lesion. Sci. Rep. 11:12743. doi: 10.1038/s41598-021-92027-z, PMID: 34140572 PMC8211659

[ref9] Bi L. Triadafilopoulos G. (2003). Exercise and gastrointestinal function and disease: an evidence-based review of risks and benefits. Clin. Gastroenterol. Hepatol. 1, 345–355. doi: 10.1053/s1542-3565(03)00178-2, PMID: 15017652

[ref10] Brinkemper A. Grasmücke D. Yilmaz E. Reinecke F. Schildhauer T. A. Aach M. (2023). Influence of locomotion therapy with the wearable cyborg HAL on bladder and bowel function in acute and chronic SCI patients. Global Spine J. 13, 668–676. doi: 10.1177/21925682211003851, PMID: 33858209 PMC10240584

[ref11] Browning K. N. Travagli R. A. (2014). Central nervous system control of gastrointestinal motility and secretion and modulation of gastrointestinal functions. Compr. Physiol. 4, 1339–1368. doi: 10.1002/cphy.c130055, PMID: 25428846 PMC4858318

[ref12] Cao H. Liu X. An Y. Zhou G. Liu Y. Xu M.. (2017). Dysbiosis contributes to chronic constipation development via regulation of serotonin transporter in the intestine. Sci. Rep. 7:10322. doi: 10.1038/s41598-017-10835-8, PMID: 28871143 PMC5583244

[ref13] Chun A. Asselin P. K. Knezevic S. Kornfeld S. Bauman W. A. Korsten M. A.. (2020). Changes in bowel function following exoskeletal-assisted walking in persons with spinal cord injury: an observational pilot study. Spinal Cord 58, 459–466. doi: 10.1038/s41393-019-0392-z, PMID: 31822808 PMC7145720

[ref14] Côté M. P. Murray M. Lemay M. A. (2017). Rehabilitation strategies after spinal cord injury: inquiry into the mechanisms of success and failure. J. Neurotrauma 34, 1841–1857. doi: 10.1089/neu.2016.4577, PMID: 27762657 PMC5444418

[ref15] de Schryver A. M. Keulemans Y. C. Peters H. P. Akkermans L. M. Smout A. J. de Vries W. R.. (2005). Effects of regular physical activity on defecation pattern in middle-aged patients complaining of chronic constipation. Scand. J. Gastroenterol. 40, 422–429. doi: 10.1080/00365520510011641, PMID: 16028436

[ref16] Dimidi E. Christodoulides S. Scott S. M. Whelan K. (2017). Mechanisms of action of probiotics and the gastrointestinal microbiota on gut motility and constipation. Adv. Nutr. 8, 484–494. doi: 10.3945/an.116.014407, PMID: 28507013 PMC5421123

[ref17] Du J. Zayed A. A. Kigerl K. A. Zane K. Sullivan M. B. Popovich P. G. (2021). Spinal cord injury changes the structure and functional potential of gut bacterial and viral communities. mSystems. 6:e01356-20. doi: 10.1128/mSystems.01356-20, PMID: 33975974 PMC8125080

[ref18] El-Salhy M. (2023). Intestinal bacteria associated with irritable bowel syndrome and chronic fatigue. Neurogastroenterol. Motil. 35:e14621. doi: 10.1111/nmo.14621, PMID: 37246923

[ref19] Everard A. Belzer C. Geurts L. Ouwerkerk J. P. Druart C. Bindels L. B.. (2013). Cross-talk between *Akkermansia muciniphila* and intestinal epithelium controls diet-induced obesity. Proc. Natl. Acad. Sci. USA 110, 9066–9071. doi: 10.1073/pnas.1219451110, PMID: 23671105 PMC3670398

[ref20] Fouad K. Popovich P. G. Kopp M. A. Schwab J. M. (2021). The neuroanatomical-functional paradox in spinal cord injury. Nat. Rev. Neurol. 17, 53–62. doi: 10.1038/s41582-020-00436-x, PMID: 33311711 PMC9012488

[ref21] Fu R. Li Z. Zhou R. Li C. Shao S. Li J. (2021). The mechanism of intestinal flora dysregulation mediated by intestinal bacterial biofilm to induce constipation. Bioengineered. 12, 6484–6498. doi: 10.1080/21655979.2021.1973356, PMID: 34517785 PMC8806846

[ref22] Gao R. Tao Y. Zhou C. Li J. Wang X. Chen L.. (2019). Exercise therapy in patients with constipation: a systematic review and meta-analysis of randomized controlled trials. Scand. J. Gastroenterol. 54, 169–177. doi: 10.1080/00365521.2019.1568544, PMID: 30843436

[ref23] GBD 2016 Traumatic Brain Injury and Spinal Cord Injury Collaborators (2019). Global, regional, and national burden of traumatic brain injury and spinal cord injury, 1990–2016: a systematic analysis for the global burden of disease study 2016. Lancet Neurol. 18, 56–87. doi: 10.1016/S1474-4422(18)30415-0, PMID: 30497965 PMC6291456

[ref9001] Ge X. Zhao W. Ding C. Tian H. Xu L. Wang H.. (2017). Potential role of fecal microbiota from patients with slow transit constipation in the regulation of gastrointestinal motility. Sci. Rep. 7:441. doi: 10.1038/s41598-017-00612-y 28348415 PMC5428802

[ref24] Glickman S. Kamm M. A. (1996). Bowel dysfunction in spinal-cord-injury patients. Lancet 347, 1651–1653. doi: 10.1016/s0140-6736(96)91487-7 8642958

[ref25] Gorgey A. S. Trainer R. Sutor T. W. Goldsmith J. A. Alazzam A. Goetz L. L.. (2021). A case study of percutaneous epidural stimulation to enable motor control in two men after spinal cord injury. Nat. Commun. 14:2064. doi: 10.1038/s41467-023-37845-7, PMID: 37045845 PMC10091329

[ref26] Gorgey A. S. Wade R. Sumrell R. Villadelgado L. Khalil R. E. Lavis T. (2017). Exoskeleton training May improve level of physical activity after spinal cord injury: A case series. Top Spinal Cord Inj Rehabil. 23, 245–255. doi: 10.1310/sci16-00025, PMID: 29339900 PMC5562032

[ref27] Gorman P. H. Forrest G. F. Asselin P. K. Scott W. Kornfeld S. Hong E.. (2021). The effect of exoskeletal-assisted walking on spinal cord injury bowel function: results from a randomised trial and comparison to other physical interventions. J. Clin. Med. 10:964. doi: 10.3390/jcm10050964, PMID: 33801165 PMC7957745

[ref28] Gungor B. Adiguzel E. Gursel I. Yilmaz B. Gursel M. (2016). Intestinal microbiota in patients with spinal cord injury. PLoS One 11:e0145878. doi: 10.1371/journal.pone.0145878, PMID: 26752409 PMC4709077

[ref29] Guo Y. Song L. Huang Y. Li X. Xiao Y. Wang Z.. (2022). *Latilactobacillus sakei* Furu2019 and stachyose as probiotics, prebiotics, and synbiotics alleviate constipation in mice. Front. Nutr. 9:1039403. doi: 10.3389/fnut.2022.1039403, PMID: 36687730 PMC9849682

[ref30] Guo M. Yao J. Yang F. Liu W. Bai H. Ma J.. (2020). The composition of intestinal microbiota and its association with functional constipation of the elderly patients. Future Microbiol. 15, 163–175. doi: 10.2217/fmb-2019-0283, PMID: 32079430

[ref31] Henke A. M. Billington Z. J. Gater D. R. Jr. (2022). Autonomic dysfunction and management after spinal cord injury: a narrative review. J Pers Med. 12:1110. doi: 10.3390/jpm12071110, PMID: 35887607 PMC9320320

[ref32] Hubscher C. H. Herrity A. N. Williams C. S. Montgomery L. R. Willhite A. M. Angeli C. A.. (2018). Improvements in bladder, bowel and sexual outcomes following task-specific locomotor training in human spinal cord injury. PLoS One 13:e0190998. doi: 10.1371/journal.pone.0190998, PMID: 29385166 PMC5791974

[ref33] Inskip J. A. Lucci V. M. McGrath M. S. Willms R. Claydon V. E. (2018). A community perspective on bowel management and quality of life after spinal cord injury: the influence of autonomic dysreflexia. J. Neurotrauma 35, 1091–1105. doi: 10.1089/neu.2017.5343, PMID: 29239268 PMC5908418

[ref34] Kigerl K. A. Hall J. C. Wang L. Mo X. Yu Z. Popovich P. G. (2016). Gut dysbiosis impairs recovery after spinal cord injury. J. Exp. Med. 213, 2603–2620. doi: 10.1084/jem.20151345, PMID: 27810921 PMC5110012

[ref35] Kim S. E. Choi S. C. Park K. S. Park M. I. Shin J. E. Lee T. H.. (2015). Change of fecal flora and effectiveness of the short-term VSL#3 probiotic treatment in patients with functional constipation. J. Neurogastroenterol. Motil. 21, 111–120. doi: 10.5056/jnm14048, PMID: 25537674 PMC4288088

[ref36] Kim H. S. Park J. H. Lee H. S. Lee J. Y. Jung J. W. Park S. B.. (2021). Effects of wearable powered exoskeletal training on functional mobility, physiological health and quality of life in non-ambulatory spinal cord injury patients. J. Korean Med. Sci. 36:e80. doi: 10.3346/jkms.2021.36.e80, PMID: 33783145 PMC8007419

[ref37] Korecka A. Dona A. Lahiri S. Tett A. J. al-Asmakh M. Braniste V.. (2016). Bidirectional communication between the aryl hydrocarbon receptor (AhR) and the microbiome tunes host metabolism. NPJ Biofilms Microbiomes. 2:16014. doi: 10.1038/npjbiofilms.2016.14, PMID: 28721249 PMC5515264

[ref38] Kwok S. Harvey L. Glinsky J. Bowden J. L. Coggrave M. Tussler D. (2015). Does regular standing improve bowel function in people with spinal cord injury? A randomised crossover trial. Spinal Cord. 53, 36–41. doi: 10.1038/sc.2014.189, PMID: 25366527

[ref39] Lensu S. Pekkala S. (2021). Gut microbiota, microbial metabolites and human physical performance. Meta 11:716. doi: 10.3390/metabo11110716, PMID: 34822374 PMC8619554

[ref40] Li J. Lin S. Vanhoutte P. M. Woo C. W. Xu A. (2016). *Akkermansia muciniphila* protects against atherosclerosis by preventing metabolic endotoxemia-induced inflammation in Apoe−/− mice. Circulation 133, 2434–2446. doi: 10.1161/CIRCULATIONAHA.115.019645, PMID: 27143680

[ref41] Liu C. W. Huang C. C. Chen C. H. Yang Y. H. Chen T. W. Huang M. H. (2010). Prediction of severe neurogenic bowel dysfunction in persons with spinal cord injury. Spinal Cord 48, 554–559. doi: 10.1038/sc.2009.181, PMID: 20065986

[ref42] Lo C. Tran Y. Anderson K. Craig A. Middleton J. (2016). Functional priorities in persons with spinal cord injury: using discrete choice experiments to determine preferences. J. Neurotrauma 33, 1958–1968. doi: 10.1089/neu.2016.4423, PMID: 27080545

[ref43] Loranskaia I. D. Boldyreva M. N. Lavrent’Eva O. A. (2013). The composition of the gastrointestinal mucosa-associated microbiota in irritable bowel syndrome patients. Eksp. Klin. Gastroenterol. 3, 15–22. 24294767

[ref44] Lynch A. C. Frizelle F. A. (2006). Colorectal motility and defecation after spinal cord injury in humans. Prog. Brain Res. 152, 335–343. doi: 10.1016/S0079-6123(05)52022-3, PMID: 16198711

[ref45] Ma T. Yang N. Xie Y. Li Y. Xiao Q. Li Q.. (2023). Effect of the probiotic strain, *Lactiplantibacillus plantarum* P9, on chronic constipation: a randomized, double-blind, placebo-controlled study. Pharmacol. Res. 191:106755. doi: 10.1016/j.phrs.2023.106755, PMID: 37019193

[ref46] Macchione I. G. Lopetuso L. R. Ianiro G. Napoli M. Gibiino G. Rizzatti G.. (2019). *Akkermansia muciniphila*: key player in metabolic and gastrointestinal disorders. Eur. Rev. Med. Pharmacol. Sci. 23, 8075–8083. doi: 10.26355/eurrev_201909_19024, PMID: 31599433

[ref47] Macpherson A. J. Pachnis V. Prinz M. (2023). Boundaries and integration between microbiota, the nervous system, and immunity. Immunity 56, 1712–1726. doi: 10.1016/j.immuni.2023.07.011, PMID: 37557080

[ref48] Mancabelli L. Milani C. Lugli G. A. Turroni F. Mangifesta M. Viappiani A.. (2017). Unveiling the gut microbiota composition and functionality associated with constipation through metagenomic analyses. Sci. Rep. 7:9879. doi: 10.1038/s41598-017-10663-w, PMID: 28852182 PMC5575163

[ref49] Moitinho-Silva L. Wegener M. May S. Schrinner F. Akhtar A. Boysen T. J.. (2021). Short-term physical exercise impacts on the human holobiont obtained by a randomised intervention study. BMC Microbiol. 21:162. doi: 10.1186/s12866-021-02214-1, PMID: 34078289 PMC8170780

[ref9002] Mokhtarzade M. Molanouri S. M. Abolhasani M. Bakhshi B. Sahraian M. A. Quinn L. S.. (2021). Home-based exercise training influences gut bacterial levels in multiple sclerosis. Complement Ther Clin Pract. 45:101463. doi: 10.1016/j.ctcp.2021.101463 34348201

[ref50] Munukka E. Ahtiainen J. P. Puigbó P. Jalkanen S. Pahkala K. Keskitalo A.. (2018). Six-week endurance exercise alters gut metagenome that is not reflected in systemic metabolism in over-weight women. Front. Microbiol. 9:2323. doi: 10.3389/fmicb.2018.02323, PMID: 30337914 PMC6178902

[ref51] Obata Y. Castaño Á. Boeing S. Bon-Frauches A. C. Fung C. Fallesen T.. (2020). Neuronal programming by microbiota regulates intestinal physiology. Nature 578, 284–289. doi: 10.1038/s41586-020-1975-8, PMID: 32025031

[ref52] Parthasarathy G. Chen J. Chen X. Chia N. O'Connor H. M. Wolf P. G.. (2016). Relationship between microbiota of the colonic mucosa vs feces and symptoms, colonic transit, and methane production in female patients with chronic constipation. Gastroenterology 150, 367–79.e1. doi: 10.1053/j.gastro.2015.10.005, PMID: 26460205 PMC4727996

[ref53] Peters H. P. De Vries W. R. VanBerge-Henegouwen G. P. Akkermans L. M. (2001). Potential benefits and hazards of physical activity and exercise on the gastrointestinal tract. Gut 48, 435–439. doi: 10.1136/gut.48.3.435, PMID: 11171839 PMC1760153

[ref54] Qi Z. Middleton J. W. Malcolm A. (2018). Bowel dysfunction in spinal cord injury. Curr. Gastroenterol. Rep. 20:47. doi: 10.1007/s11894-018-0655-4 30159690

[ref55] Quiroga R. Nistal E. Estébanez B. Porras D. Juárez-Fernández M. Martínez-Flórez S.. (2020). Exercise training modulates the gut microbiota profile and impairs inflammatory signaling pathways in obese children. Exp. Mol. Med. 52, 1048–1061. doi: 10.1038/s12276-020-0459-0, PMID: 32624568 PMC8080668

[ref56] Reigstad C. S. Salmonson C. E. III J. F. R. Szurszewski J. H. Linden D. R. Sonnenburg J. L.. (2015). Gut microbes promote colonic serotonin production through an effect of short-chain fatty acids on enterochromaffin cells. FASEB J. 29, 1395–1403. doi: 10.1096/fj.14-259598, PMID: 25550456 PMC4396604

[ref57] Resende A. S. Leite G. S. F. Lancha Junior A. H. (2021). Changes in the gut Bacteria composition of healthy men with the same nutritional profile undergoing 10-week aerobic exercise training: a randomized controlled trial. Nutrients 13:2839. doi: 10.3390/nu13082839, PMID: 34444999 PMC8398245

[ref58] Rosario D. Bidkhori G. Lee S. Bedarf J. Hildebrand F. le Chatelier E.. (2021). Systematic analysis of gut microbiome reveals the role of bacterial folate and homocysteine metabolism in Parkinson’s disease. Cell Rep. 34:108807. doi: 10.1016/j.celrep.2021.108807, PMID: 33657381

[ref59] Schiering C. Wincent E. Metidji A. Iseppon A. Li Y. Potocnik A. J.. (2017). Feedback control of AHR signalling regulates intestinal immunity. Nature 542, 242–245. doi: 10.1038/nature21080, PMID: 28146477 PMC5302159

[ref60] Simpson L. A. Eng J. J. Hsieh J. T. Wolfe D. L. Spinal Cord Injury Rehabilitation Evidence Scire Research Team (2012). The health and life priorities of individuals with spinal cord injury: a systematic review. J. Neurotrauma 29, 1548–1555. doi: 10.1089/neu.2011.2226, PMID: 22320160 PMC3501530

[ref61] Sohail M. U. Yassine H. M. Sohail A. Thani A. A. A. (2019). Impact of physical exercise on gut microbiome, inflammation, and the pathobiology of metabolic disorders. Rev. Diabet. Stud. 15, 35–48. doi: 10.1900/RDS.2019.15.35 31380886 PMC6760895

[ref62] Sun W. Guo Y. Zhang S. Chen Z. Wu K. Liu Q.. (2018). Fecal microbiota transplantation can alleviate gastrointestinal transit in rats with high-fat diet-induced obesity via regulation of serotonin biosynthesis. Biomed. Res. Int. 2018:8308671. doi: 10.1155/2018/8308671, PMID: 30370307 PMC6189652

[ref63] Takeda T. Asaoka D. Nojiri S. Yanagisawa N. Nishizaki Y. Osada T.. (2023). Usefulness of *Bifidobacterium longum* BB536 in elderly individuals with chronic constipation: A randomized controlled trial. Am. J. Gastroenterol. 118, 561–568. doi: 10.14309/ajg.0000000000002028, PMID: 36216361 PMC9973440

[ref64] Tate D. G. Forchheimer M. Rodriguez G. Chiodo A. Cameron A. P. Meade M.. (2016). Risk factors associated with neurogenic bowel complications and dysfunction in spinal cord injury. Arch. Phys. Med. Rehabil. 97, 1679–1686. doi: 10.1016/j.apmr.2016.03.019, PMID: 27109330

[ref65] Vallès M. Mearin F. (2009). Pathophysiology of bowel dysfunction in patients with motor incomplete spinal cord injury: comparison with patients with motor complete spinal cord injury. Dis. Colon Rectum 52, 1589–1597. doi: 10.1007/DCR.0b013e3181a873f3, PMID: 19690487

[ref66] Vallès M. Vidal J. Clavé P. Mearin F. (2006). Bowel dysfunction in patients with motor complete spinal cord injury: clinical, neurological, and pathophysiological associations. Am. J. Gastroenterol. 101, 2290–2299. doi: 10.1111/j.1572-0241.2006.00729.x, PMID: 17032195

[ref67] Wang L. Hu L. Xu Q. Yin B. Fang D. Wang G.. (2017). *Bifidobacterium adolescentis* exerts strain-specific effects on constipation induced by loperamide in BALB/c mice. Int. J. Mol. Sci. 18:318. doi: 10.3390/ijms18020318, PMID: 28230723 PMC5343854

[ref68] Wang J. Wang L. Yu Q. Tang N. Mei C. Zhang H.. (2023). Characteristics of the gut microbiome and serum metabolome in patients with functional constipation. Nutrients 15:1779. doi: 10.3390/nu15071779, PMID: 37049619 PMC10097253

[ref69] Wegierska A. E. Charitos I. A. Topi S. Potenza M. A. Montagnani M. Santacroce L. (2022). The connection between physical exercise and gut microbiota: implications for competitive sports athletes. Sports Med. 52, 2355–2369. doi: 10.1007/s40279-022-01696-x, PMID: 35596883 PMC9474385

[ref70] Wortelboer K. Nieuwdorp M. Herrema H. (2019). Fecal microbiota transplantation beyond Clostridioides difficile infections. EBioMedicine 44, 716–729. doi: 10.1016/j.ebiom.2019.05.066, PMID: 31201141 PMC6606746

[ref71] Wulf M. J. Tom V. J. (2023). Consequences of spinal cord injury on the sympathetic nervous system. Front. Cell. Neurosci. 17:999253. doi: 10.3389/fncel.2023.999253, PMID: 36925966 PMC10011113

[ref72] Xiang X. N. Ding M. F. Zong H. Y. Liu Y. Cheng H. He C. Q.. (2020). The safety and feasibility of a new rehabilitation robotic exoskeleton for assisting individuals with lower extremity motor complete lesions following spinal cord injury (SCI): an observational study. Spinal Cord 58, 787–794. doi: 10.1038/s41393-020-0423-9, PMID: 32034295

[ref73] Yano J. M. Yu K. Donaldson G. P. Shastri G. G. Ann P. Ma L.. (2015). Indigenous bacteria from the gut microbiota regulate host serotonin biosynthesis. Cell 161, 264–276. doi: 10.1016/j.cell.2015.02.047, PMID: 25860609 PMC4393509

[ref74] Yu B. Qiu H. Cheng S. Ye F. Li J. Chen S.. (2021). Profile of gut microbiota in patients with traumatic thoracic spinal cord injury and its clinical implications: a case-control study in a rehabilitation setting. Bioengineered. 12, 4489–4499. doi: 10.1080/21655979.2021.1955543, PMID: 34311653 PMC8806552

[ref75] Zhang S. Wang R. Li D. Zhao L. Zhu L. (2021). Role of gut microbiota in functional constipation. Gastroenterol. Rep. (Oxf). 9, 392–401. doi: 10.1093/gastro/goab035, PMID: 34733524 PMC8560038

[ref76] Zhang C. Zhang W. Zhang J. Jing Y. Yang M. du L.. (2018). Gut microbiota dysbiosis in male patients with chronic traumatic complete spinal cord injury. J. Transl. Med. 16:353. doi: 10.1186/s12967-018-1735-9, PMID: 30545398 PMC6293533

[ref77] Zhao L. Huang Y. Lu L. Yang W. Huang T. Lin Z.. (2018). Saturated long-chain fatty acid-producing bacteria contribute to enhanced colonic motility in rats. Microbiome. 6:107. doi: 10.1186/s40168-018-0492-6, PMID: 29903041 PMC6003035

[ref78] Zheng Y. Jiang X. Gao Y. Yuan L. Wang X. Wu S.. (2021). Microbial profiles of patients with antipsychotic-related constipation treated with electroacupuncture. Front. Med. (Lausanne). 8:737713. doi: 10.3389/fmed.2021.737713, PMID: 34722577 PMC8551555

[ref79] Zhu L. Liu W. Alkhouri R. Baker R. D. Bard J. E. Quigley E. M.. (2014). Structural changes in the gut microbiome of constipated patients. Physiol. Genomics 46, 679–686. doi: 10.1152/physiolgenomics.00082.2014, PMID: 25073603

